# A pilot survey of selenium status and its geospatial variation among children and women in three rural districts of Zimbabwe

**DOI:** 10.3389/fnut.2023.1235113

**Published:** 2023-07-11

**Authors:** Beaula Mutonhodza, Christopher Chagumaira, Mavis P. Dembedza, Edward J. M. Joy, Muneta G. Manzeke-Kangara, Handrea Njovo, Tasiana K. Nyadzayo, R. Murray Lark, Alexander A. Kalimbira, Elizabeth H. Bailey, Martin R. Broadley, Tonderayi M. Matsungo, Prosper Chopera

**Affiliations:** ^1^Department of Nutrition, Dietetics and Food Sciences, University of Zimbabwe, Harare, Zimbabwe; ^2^School of Biosciences, Sutton Bonington Campus, University of Nottingham, Loughborough, United Kingdom; ^3^London School for Hygiene & Tropical Medicine, London, United Kingdom; ^4^Rothamsted Research, Harpenden, United Kingdom; ^5^National Nutrition Unit, Ministry of Health and Child Care of Zimbabwe, Harare, Zimbabwe; ^6^Department of Human Nutrition and Health, Lilongwe University of Agriculture and Natural Resources, Lilongwe, Malawi

**Keywords:** selenium deficiency, conditional kriging, geospatial patterns, micronutrients, glutathione peroxidase 3, iodothyronine deiodinase

## Abstract

**Introduction:**

Selenium (Se) deficiency is increasingly recognized as a public health problem in sub-Saharan Africa.

**Methods:**

The current cross-sectional study assessed the prevalence and geospatial patterns of Se deficiency among children aged 6–59 months (*n* = 741) and women of 15–49 years old (*n* = 831) selected by simple random sampling in rural Zimbabwe (Murewa, Shamva, and Mutasa districts). Venous blood samples were collected and stored according to World Health Organization guidelines. Plasma Se concentration was determined by inductively coupled plasma-mass spectrometry.

**Results:**

Median, Q1, and Q3 plasma Se concentrations were 61.2, 48.7, and 73.3 μg/L for women and 40.5, 31.3, and 49.5 μg/L for children, respectively. Low plasma Se concentrations (9.41 μg/L in children and 10.20 μg/L in women) indicative of severe Se deficiency risk was observed. Overall, 94.6% of children and 69.8% of women had sub-optimal Se status defined by plasma Se concentrations of <64.8 μg/L and <70 μg/L, respectively.

**Discussion:**

High and widespread Se deficiency among women and children in the three districts is of public health concern and might be prevalent in other rural districts in Zimbabwe. Geostatistical analysis by conditional kriging showed a high risk of Se deficiency and that the Se status in women and children in Murewa, Shamva, and Mutasa districts was driven by short-range variations of up to ⁓12 km. Selenium status was homogenous within each district. However, there was substantial inter-district variation, indicative of marked spatial patterns if the sampling area is scaled up. A nationwide survey that explores the extent and spatial distribution of Se deficiency is warranted.

## 1. Introduction

Worldwide, an estimated one-in-two children aged 6–59 months and two-in-three women of reproductive age (WRA) have at least one micronutrient deficiency (1). In sub-Saharan Africa (SSA) and South Asia, children aged 6–59 months and WRA are the most vulnerable to vitamin and mineral deficiencies, due in part to greater requirements (1–3). Recent estimates of dietary mineral supplies indicate that micronutrient deficiencies (MNDs), such as Se may have considerable public health significance (4, 5).

Selenium is an essential micronutrient for human health (6–8). Selenium forms inorganic and organic compounds. The inorganic forms include selenate (VI) and sodium selenite (selenate IV), while the organic forms include selenomethionine and methylated selenocysteine (9). Selenium speciation significantly affects the potential benefits of this element to mammal health, with organic Se forms (selenocysteine and/or selenomethionine) being the most effective bioavailable Se species for human nutrition (10–12). Selenium is typically found in meat and meat products (13). Negligible amounts are obtained via drinking water (14). Selenium has multiple biological activities, which depend on the level of Se intake (15). Selenium is a component of enzymes that play a key role in the regulation of thyroid hormone metabolism, antioxidant defense systems, and oxidative metabolism (8, 13). Selenium deficiencies can lead to gestational complications, miscarriages, and oxidative stress (13, 16).

Relatively low Se intakes determine the expression of selenoenzymes in which it serves as an essential constituent whereas, higher intakes have been shown to have anti-tumorigenic potential (15). Selenium exposure with beneficial effects on human health has generally been limited to around 50–100 μg/day unless dietary supplements with higher levels of Se are consumed (13, 17) with an upper tolerable limit of 400 μg/day (18). On the other hand, supra-nutritional levels might have adverse effects (19). The recommended intakes of Se have been calculated from the requirement for optimum plasma glutathione peroxidase 3 (GPX3), selenoprotein P (SELENOP), and iodothyronine deiodinase (IDI) activities based on Thomson (10). The World Health Organization (WHO) recommends a daily intake of Se at a level of 55 μg for adults (18). The Se requirements for pregnant and lactating women are higher and range from 60–70 μg/day and that for children range between 15–40 μg/day depending on age (20, 21). However, at the country level, there is a lot of variation in the recommended daily intakes (13, 22).

Human Se deficiency status is generally defined as plasma Se concentrations less than 70 μg/L (23). Various thresholds of plasma Se concentration have been used to define deficiency based on the associated expression of Se-containing proteins (10, 24). Plasma Se concentrations of >100, >84.9, and >64.8 μg/L are typically used as thresholds for optimal activities of selenoprotein P (SELENOP), glutathione peroxidase 3 (GPX3), and iodothyronine deiodinase (IDI), respectively (10, 24). Selenoprotein P is essential for Se transport and homeostasis (25), GPX3 contributes to protecting the organism from oxidative damage (9), and IDI is essential for growth and normal thyroid function (26). Selenium deficiency is associated with the pathogenicity of several viruses, including human immunodeficiency virus (HIV) (27). The African region is the most affected, with 25.7 million people living with HIV in 2018 (28). Viral infection simultaneously increases the demand for micronutrients and causes their loss, exacerbating deficiency (27). Selenium deficiency poses a potential public health concern, particularly in Africa, where HIV is most prevalent.

Worldwide, up to one in seven people have been estimated to have low dietary Se intake (29). Dietary Se deficiency is estimated to affect about 0.5 to 1 billion people across the world (30, 31) and this may be exacerbated by climate change (32). Selenium deficiency is widespread among women and children in Africa (33, 34), where a 28% prevalence of inadequate dietary Se intake was estimated (35). The Se status of populations varies markedly as a result of different geological, geochemical, and climatic factors (36). Marked spatial variations and patterns in human Se status have been observed in Malawi (24, 37) and Ethiopia (23, 38). Soil, forage, livestock, and human Se deficiencies have been previously reported in Zimbabwe (39–41). However, no national Se data exist for the country. The current study reports findings from a pilot micronutrient biomarker survey, the aims of which were to inform micronutrient national surveillance and provide a clear evidence base for the delivery of targeted interventions across Zimbabwe by the Government of Zimbabwe (GoZ), partners, and other stakeholders. The study looked at the prevalence and geospatial patterns of Se deficiency in women and children from three rural districts; Murewa, Shamva, and Mutasa.

## 2. Materials and methods

### 2.1. Study site, sampling, and participants

The current paper presents data on the prevalence and geospatial patterns of Se deficiency in WRA (*n* = 831) and children aged 6–59 months (*n* = 741) from a cross-sectional biomarker survey conducted between October 2021 and January 2022. The survey covered three rural districts; Murewa (17.6502°S, 31.7787°E), Shamva (17.04409°S, 31.6739°E), and Mutasa (18.6155°S, 32.6730°E) in Zimbabwe. The districts were chosen based on high stunting rates at 36.2, 37.7, and 30.9%, respectively and low dietary diversity, with 15–29.9% of children 6–59 months getting the minimum number of required food groups (42, 43). The sampling design was nested at the level of the National Demographic Health Survey (DHS) sampling approach (44).

**Figure 1:**
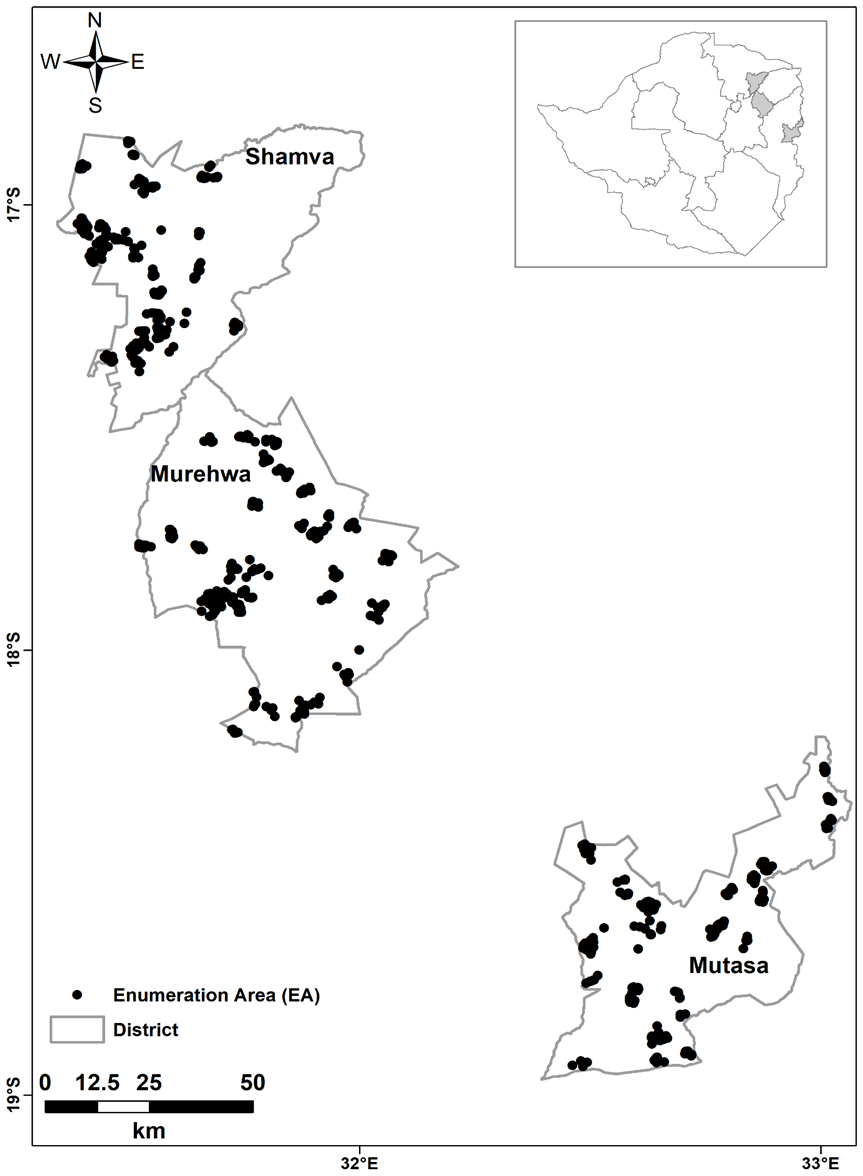
Sampling locations of eligible households (*n* = 900), from which study participants were recruited in Shamva, Murewa, and Mutasa districts from rural Zimbabwe.

Thirty Enumeration areas (EAs) were selected per district. An EA contains an average of 120 households (44). EAs were selected by proportional probability sampling (PPS) with inclusion probabilities proportional to the most recently recorded population (45). Household listing was undertaken for all eligible households in the selected EAs. Following the listing, 10 households in each EA were selected by random systematic sampling without replacement. In households where more than one set of eligible individuals was identified, one mother–child pair was randomly selected using the Kish Grid (46). The location of each household (sampling point) was determined using a global positioning system receiver (GPS) and verified through matched EA shape files (Figure 1). Recruitment was done at the household level, with participants then directed to the nearest health facility for data collection. Additional information on the sampling design is described in Mutonhodza et al. (70).

### 2.2. Data collection and analysis

#### 2.2.1. Demographic characteristics

A questionnaire adapted from the ZDHS was used to collect household demographic data (44). This questionnaire assessed residency by district, maternal age, child age, and sex amongst other parameters. Enumeration was done by trained research assistants from the Ministry of Health and Child Care (MoHCC) and the University of Zimbabwe. The questionnaire was standardized in the local language, pilot tested, and refined before data collection.

#### 2.2.2. Data and sample management

A temporary laboratory was established at each collection site to minimize contamination, facilitate accurate record keeping, and for traceability of samples. Strict quality control measures were followed as guided by the Centre for Disease Control and Prevention (CDC) (47). All participants were assigned a unique numeric ID that was labeled on samples, the labels were identifiable by district, demographic group, and destination. The IDs were also used on data capture forms, sample collection materials, and subsequent analyses to maintain anonymity. Demographic and specimen data were collected using passcode-protected tablets with KoboToolbox software (Android v2022.1.2). Data was reconciled and transferred daily to the central data processing server.

#### 2.2.3. Blood sampling and analysis

One casual blood sample (6 mL blood) was collected from WRA and children 6–59 months by venipuncture by experienced phlebotomists and processed as per the World Health Organization (WHO) blood collection guidelines (48). Blood was centrifuged using a portable centrifuge model 521-2,854 (VWR compactStar CS4, Rotor. United Kingdom) to isolate the plasma and aliquoted in triplicate into cryovials in the field. CDC guidelines were followed to reduce the risk of hemolysis (49). Plasma samples were transported daily from the collection health centers to the district hospital, via a cold chain before being taken to the University of Zimbabwe laboratory in Harare where they were stored at −80°C. One replicate sample of plasma was shipped on dry ice to Germany for analysis of biomarkers of inflammation, CRP, and AGP by a sandwich ELISA as adapted from Erhardt et al. (71).

Another set of replicate plasma samples was shipped on dry ice to the University of Nottingham, UK for quantification of plasma Se concentration using ICP-MS as described by Belay et al. (2020) and Phiri et al. (2019). The limit of detection (LOD) for all elements was measured as 3× the standard deviation of 10 operational blanks; the limit of quantification (LOQ) was calculated as 10× this standard deviation. The LOD and LOQ were 0.029 and 0.096 μg/L, respectively. Accuracy was verified using two appropriate reference materials (Seronorm^™^ L-1 (Lot 1,801,802) and Seronorm^™^ L-2 (Lot 1,801,803)); (Nycomed Pharma AS, Billingstad, Norway). Average Se recovery when compared to accredited values determined across 40 analytical batches of blood plasma was 99 and 102% for L-1 and L-2, respectively. There was no significant negative correlation between plasma Se concentration and any of the inflammation biomarkers (Supplementary Material 1), thus no correction was applied for inflammation (48, 50).

#### 2.2.4. Data analysis

Prevalence of Se deficiency was then compared against plasma Se concentration thresholds of 70 μg/L (23, 24) and >100, >84.9, and >64.8 μg/L previously indicated as optimal activities of SELENOP, GPX3, and IDI, respectively (10). Descriptive statistical analyses were conducted using SPSS version 20 (IBM, New York, United States), while geostatistical analyses were conducted using R (51). Exploratory data analysis used simple summary statistics and plots, notably quantile–quantile (QQ) plots to check for outliers and data normality (52). To check for evidence of spatial trends, classified post plots were plotted, which showed the spatial location of data using symbols to indicate quantiles. The variance parameters were estimated by residual maximum likelihood (REML), with the likfit (53) procedure for the R platform (51). A linear mixed model (LMM) approach was applied in which the only fixed effect was a constant mean. Following the exploratory analysis, ordinary kriging was used to predict the plasma Se concentration of unsampled WRA and children aged 6–59 months at the household level in the three districts. The predictions were made using the geoR library in the R platform (51, 53). Cross-validation of the models was performed by omission of one of the observed data points in turn from the set of Se data and its value predicted by ordinary kriging with a suitable model (54). The goodness of fit of a model was assessed by examining the median standardized squared prediction error (SSPE) (55). The exponential and spherical models were both plausible.

To map plasma Se concentrations for WRA and children, ordinary kriging estimates of individual concentrations were computed by district boundaries at nodes of a 250 m square grid. Congruent with other studies (23, 24) the conditional threshold used was: plasma Se concentration for optimal activity of GPX3 for WRA based on its reproductive functions (16) and plasma Se concentration for optimal activity of IDI for children based on its importance in child growth and development (26, 56). The conditional probabilities were presented as calibrated verbal phrases on the Intergovernmental Panel for Climate Change (IPCC) scale, supplemented with a definition of the probability range (57).

#### 2.2.5. Ethical statements

The study was conducted in line with the Declaration of Helsinki. Ethical approval was obtained from the Institutional Review Boards (IRBs) of the University of Nottingham (Reference#446-1912) and the Medical Research Council of Zimbabwe (MRCZ/A/2575 & MRCZ/A/2664). Shipping permissions, including a material transfer agreement (MTA), were secured in Zimbabwe and UK before sample shipping. Permission to conduct the research study in communities was obtained through consultative engagement with local government officials and the Ministry of Health at the provincial, district, clinic, and village levels. Written informed consent from all WRA and assent for all child participants was obtained before the commencement of data collection.

## 3. Results

From the targeted sample size of 1800 plasma Se samples (WRA, *n* = 900; children 6–59 months, *n* = 900) a total of 1,572 (WRA, *n* = 831; children 6–59 months, *n* = 741) plasma Se measurements were adequate for presentation in this paper.

### 3.1. Demography of study participants

The sample size of the three districts (Mutasa/Murewa/Shamva) was proportionate across the two demographic groups. The male/female ratio of the sampled population of children was also proportionate (1,1) across districts (Table 1). The median (Q1, Q3) age for the children was 28.8 months (17.8, 43.5), and that for women was 30 years (24, 37).

**Table 1:** Characteristics of study participants.

Characteristic	*n*	%
Children 6–59 months		
District		
Murewa	247	29.7
Shamva	274	37.0
Mutasa	220	33.3
Sex		
Male	368	49.7
Female	373	50.3
Age (months)		
Median (Q1, Q3)	28.8 (17.8, 43.5)	
Women 15–49 years		
District		
Murewa	281	30.0
Shamva	301	36.2
Mutasa	249	33.8
Age (years)		
Median (Q1, Q3)	30 (24, 37)	

### 3.2. Prevalence of Se deficiency

Selenium deficiency was highly prevalent in both women and children in all three rural districts. Children were more likely to be deficient than women, with 94.6% (<64.8 μg/L) and 69.8% (<70 μg/L), respectively. The overall median (Q1, Q3) Se concentrations were 61.2 μg/L (48.7, 73.3) and 40.5 μg/L (31.3, 49.5), respectively. Notable evidence of severe Se deficiency risk was observed in the sample population, with children recording plasma Se concentrations as low as 9.41 μg/L and 10.20 μg/L being the minimum in WRA (Table 2). Plasma Se concentrations differed between districts, ranging from a median (Q1, Q3) of 36.5 μg/L (27.8, 44.1) in Murewa district to 71.7 μg/L (62.1, 84.9) in Mutasa district. Variations in plasma Se concentrations were notable between districts. Shamva and Murewa had comparable values. Participants in Murewa district had the lowest median plasma Se concentration and participants in Mutasa had the highest. Remarkably, in Mutasa, there was a substantial difference in plasma Se concentrations between the two demographic groups with median plasma Se concentrations of 71.7 μg/L (62.1, 84.9) in WRA and 47.2 μg/L (37.1, 58.0) in children.

**Table 2:** Selenium status by district and demographic group.

Variable	District	Plasma Se (μg/L)^†^					Prevalence (%) of Se deficiency			
		Median	Mean	SD	Min.	Max.	Plasma Se^‡^ (<70 μg/L)	SELENOP (<100 μg/L)	GPX3 (<84.9 μg/L)	IDI (<64.8 μg/L)
Children 6–59 months										
	Murewa	36.5 (27.8, 44.1)	37.0	12.6	9.4	80.0	98.0	100	100	97.6
	Shamva	40.2 (31.6, 48.0)	40.1	11.3	12.2	76.8	98.2	100	100	97.8
	Mutasa	47.2 (37.1, 58.0)	48.5	18.5	12.7	175.2	92.3	98.2	96.8	87.3
	Overall	40.5 (31.3, 49.5)	41.6	15.0	9.4	175.2	96.4	99.5	99.1	94.6
Women 15–49 years										
	Murewa	53.0 (42.5, 64.8)	53.8	16.7	10.2	115.3	84.0	98.9	96.8	74.8
	Shamva	58.5 (47.6, 70.0)	59.6	17.7	17.4	150.4	75.1	98.7	92.7	61.1
	Mutasa	71.7 (62.1, 84.9)	74.3	21.3	27.9	184.3	47.4	91.6	75.5	31.7
	Overall	61.2 (48.7, 73.3)	62.0	20.3	10.2	184.3	69.8	96.6	88.9	57.0

^†^Values in brackets are Q1: 25th percentile and Q3: 75th percentile, SD, standard deviation; Min., minimum value; Max., maximum value. ‡Plasma Se concentration is the standard reference for Se status determination, SELENOP, Selenoprotein P; GPX3, glutathione peroxidase 3; IDI, iodothyronine deiodinase.

### 3.3. Geostatistical modeling of Se deficiency in WRA and children 6–59 months in Shamva, Murewa, and Mutasa districts

#### 3.3.1. Exploratory analysis

Of the 1,572 plasma samples analysed 183 (WRA = 74, children = 109) were excluded from the geostatistical analysis due to erroneous or missing GPS coordinates (23), to improve the accuracy of the variogram estimations. The assumption was that the missing data would not introduce bias as the minimum number of sampling points used (200) exceeded the minimum required for effective variogram estimations (54). A few outliers were observed in the upper tail and were accounted for by the use of the Cressie-Hawkins (58), a robust estimator which downplays the effect of outliers (54). All the variables were regarded as normally distributed (Table 3) as the Se data did not have significantly skewed distributions (octile skew <0.2) (59), suggesting that the assumption of stationary variation about a fixed mean was reasonable (55). From the classified post plots, there was no visible spatial trend in the distribution of plasma Se concentrations in both women and children in all three districts.

**Table 3:** Geospatial exploratory analysis of plasma Se data for WRA and Children in Mutasa, Shamva, and Murewa districts.

Variable	District	# of data^†^	Mean	Median	Q1	Q3	Variance	SD	Octile skewness
Children 6–59 months									
	Murewa	208	37	36.5	27.9	44.1	156.9	12.5	0.03
	Shamva	223	40.1	40.2	31.6	47.9	129.3	11.4	−0.06
	Mutasa	201	48.5	47.2	37.1	57.6	342.1	18.5	0.04
Women 15–49 years									
	Murewa	258	53.9	53.0	42.6	64.8	279	16.7	0.05
	Shamva	260	59.1	58.4	47.5	69.5	290	17.0	0.03
	Mutasa	239	74.7	71.8	62.2	85.1	470	21.7	0.13

^†^Represents the number of sampling points with valid geo-coordinates used for geostatistical analysis.

#### 3.3.2. Variogram estimation and validation

Table 4 shows the median standardized squared prediction error (SSPE) for the cross-validation of each variogram model. The variogram model selected in each case was the one with a median SSPE closest to the expected value of 0.455 for a valid variogram model (55, 60). The variogram model was selected in each case based on the model that had the greatest median SSPE value, within the 95% confidence interval.

**Table 4:** Goodness of fit of covariance models by cross-validation.

Covariance model	Median SSPE		
	Murewa	Shamva	Mutasa
Children 6–59 months			
	*n* = 208	*n* = 223	*n* = 201
Exponential	0.431	0.455	0.323
Spherical	0.432	0.439	0.342
Women of reproductive age			
	*n* = 258	*n* = 260	*n* = 239
Exponential	0.416	0.381	0.309
Spherical	0.406	0.376	0.295

The confidence interval for the median SSPE for the children biomarker (all districts) is (0.31, 0.60) and for WRA it is (0.32, 0.59).

There were some differences among the districts with respect to the spatial variation of plasma Se. In Murewa district, the correlated variance was largest relative to the uncorrelated variance (intercept of the variogram function) for both demographic groups, with spatial dependence of 5 to 7 km (Supplementary Material 2). In Shamva district the spatially correlated variance was smaller relative to the uncorrelated variance, the range of spatial correlation was short for WRA (less than 5 km) and longer for children (around 12 km). In Mutasa district, the behavior was intermediate between the other two with spatial correlation at similar scales to those seen in Murewa (Supplementary Material 2).

#### 3.3.3. Ordinary kriging prediction

**Figure 2:**
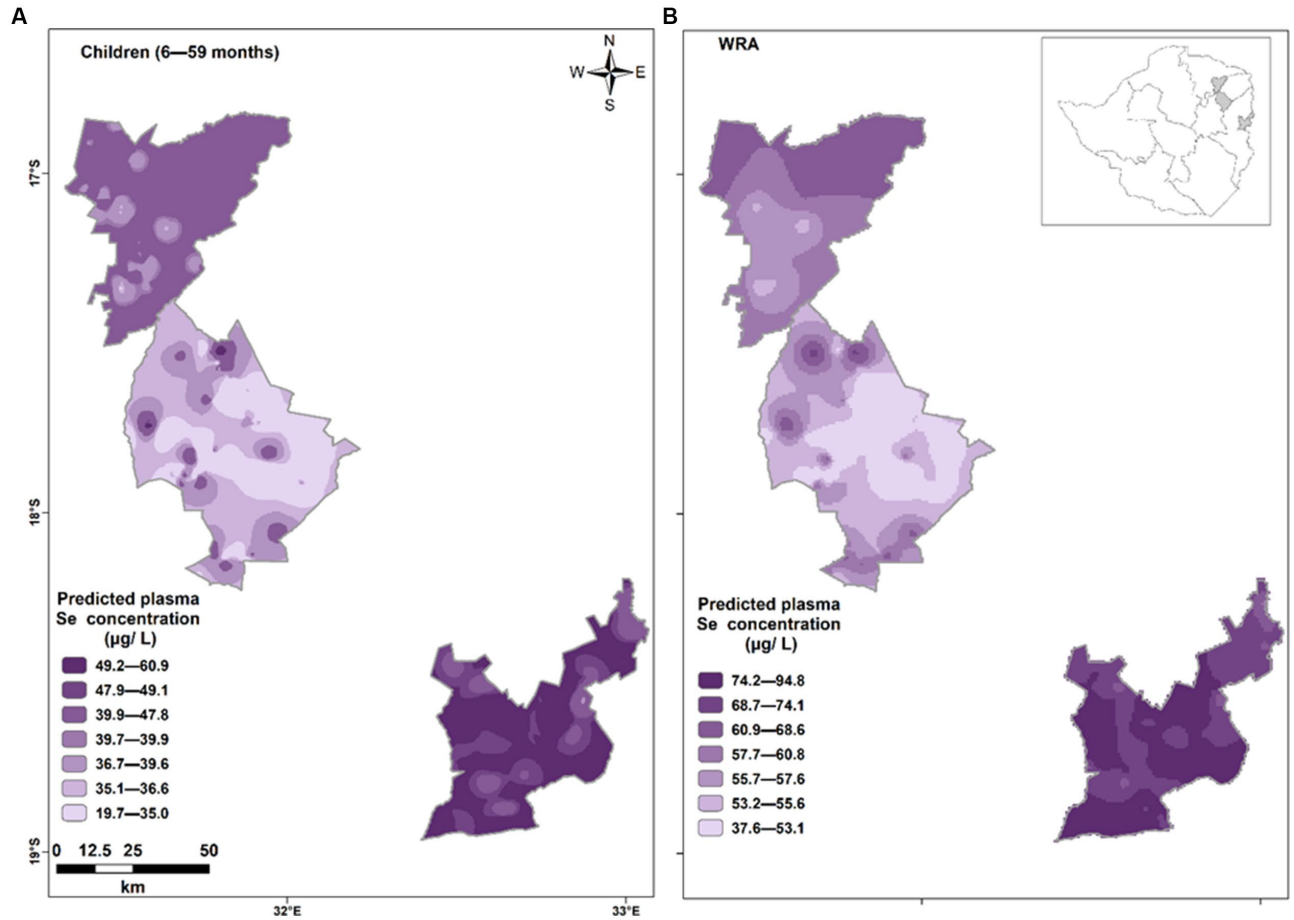
Predicted plasma Se concentration in **(A)** children 6–59 months and **(B)** WRA residing in Shamva (top), Murewa (middle), and Mutasa (bottom). At any location, the kriging prediction is the best linear unbiased prediction of Se status.

The variograms for both children and WRA do not show large marked spatial trends. Selenium status was reasonably homogenous within each district. However, there was substantial inter-district variation from a snapshot of three c.50 × 50 km areas (Figure 2). The output from this spatial analysis allows us to visualize the hot spots of Se deficiency in the three districts under study. The intensity of the purple color shades is commensurate with the plasma Se concentration. Lighter shades indicate low Se concentrations within the study population and darker shades indicate high plasma Se concentration. Murewa had the lowest plasma Se concentrations in both WRA and children while Mutasa recorded the highest values of plasma Se concentration.

#### 3.3.4. Kriging variance

**Figure 3:**
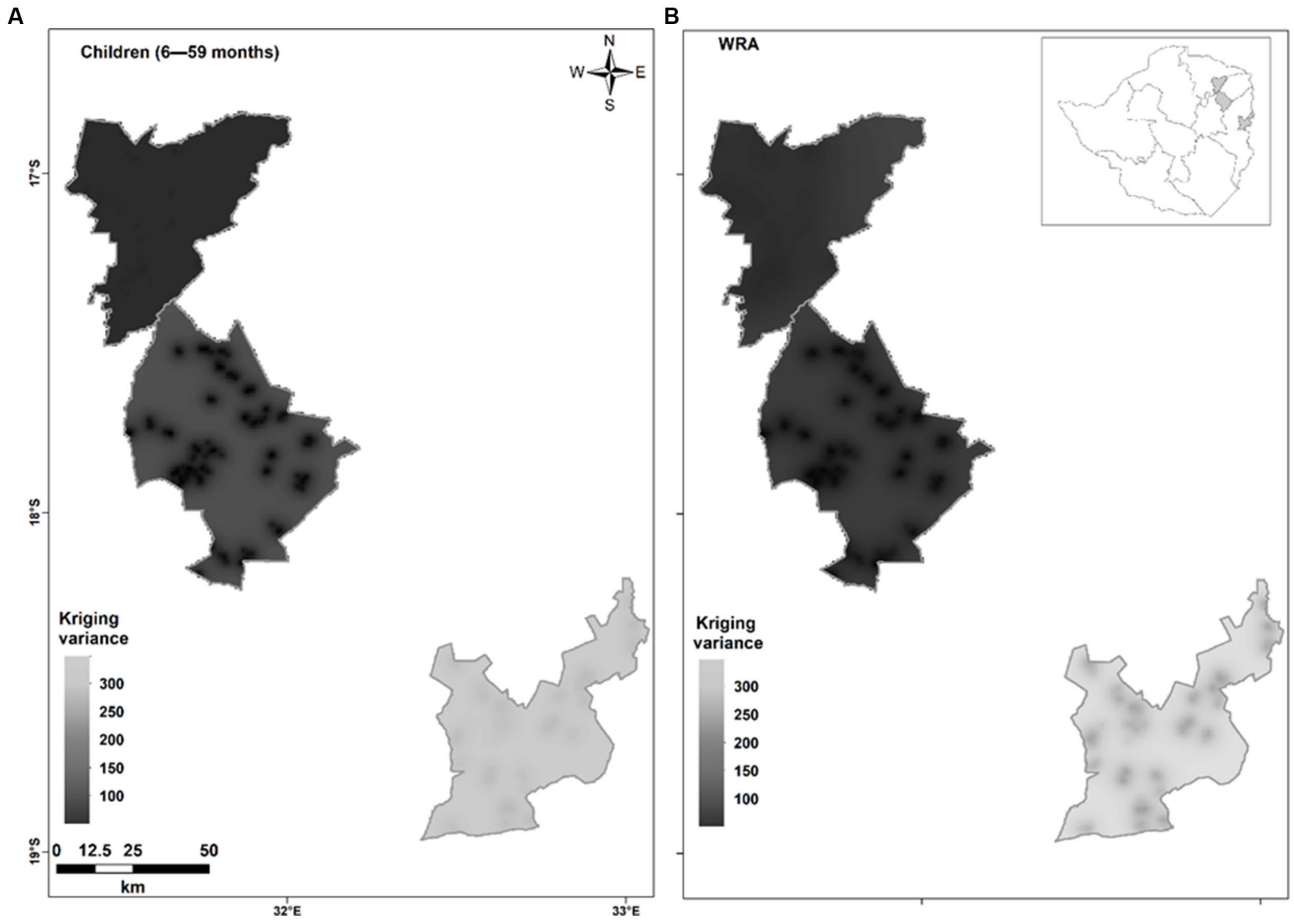
Kriging variances for plasma Se concentration in **(A)** children 6–59 months and **(B)** WRA residing in Shamva (top), Murewa (middle), and Mutasa (bottom) by ordinary kriging. The kriging variance is a measure of uncertainty of predictions at unsampled locations.

The kriging variances were high in both children 6–59 months and WRA residing in Shamva, Murewa, and Mutasa districts with average values ranging from 142–144 in children and 277–391 in WRA. Shamva had slightly lower kriging variances (Figure 3).

#### 3.3.5. Conditional probabilities

**Figure 4:**
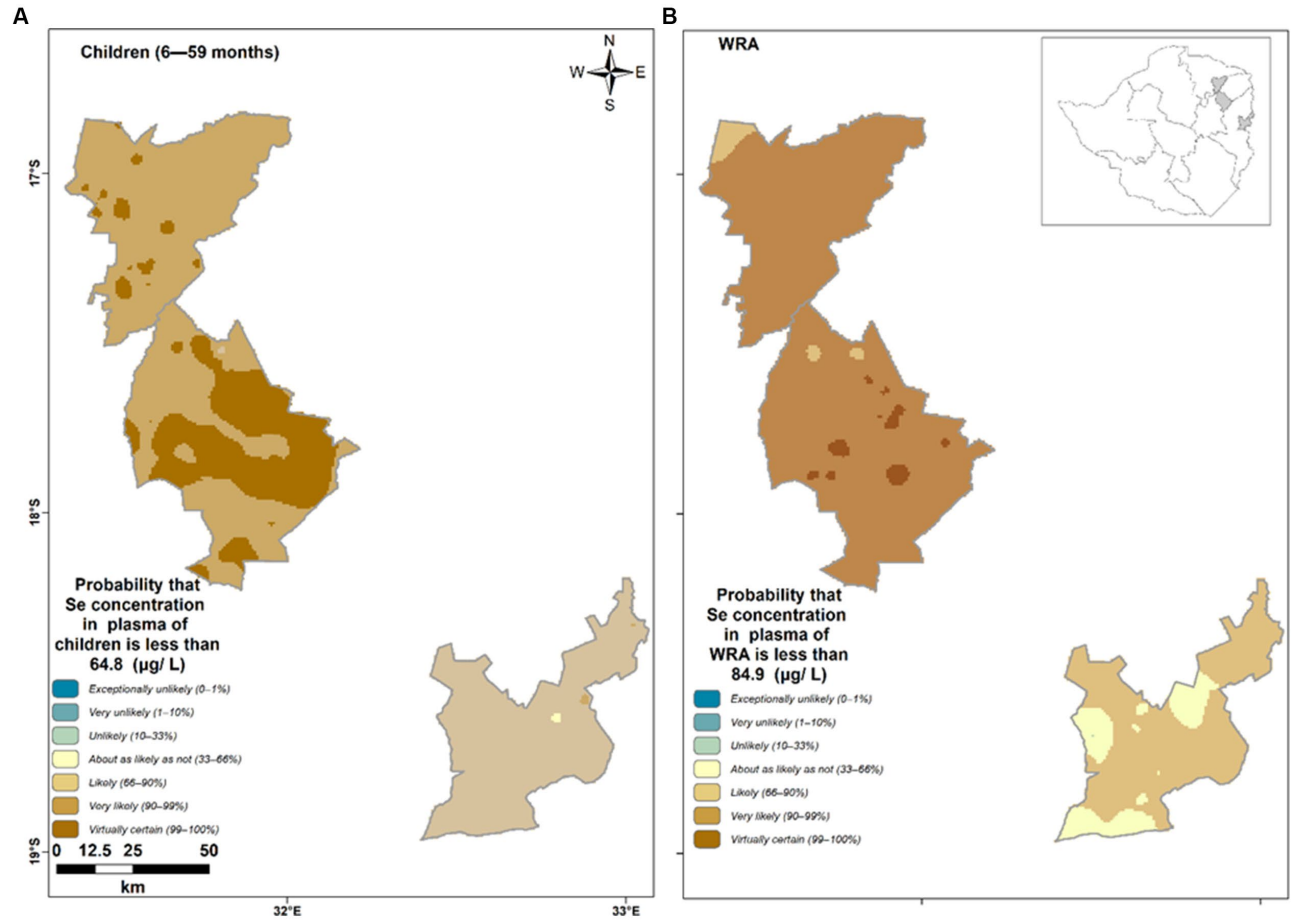
Probability that plasma Se concentration of **(A)** children falls below the threshold for the optimal activity of IDI (<64.8 μg/L) and **(B)** women of reproductive age fall below the threshold of the optimal activity of GPX3 (<84.9 μg/L) in Shamva (top), Murewa (middle), and Mutasa (bottom).

There was a 0.98, 0.98, and 0.9 average probability that the unsampled children aged 6–59 months in Murewa, Shamva, and Mutasa had plasma Se concentrations below the threshold for the optimal activity of IDI (<64.8 μg/L), respectively. Similarly, the average probability of unsampled WRA having plasma Se concentrations below the threshold of the optimal activity of GPX3 (<84.9 μg/L) was 0.97, 0.98, and 0.73 for Murewa, Shamva, and Mutasa, respectively. The verbal scale shows the likelihood of an individual being below a threshold (Figure 4). The brown color tone reflects a higher likelihood of the occurrence of Se deficiency. Children 6–59 months residing in Murewa have the highest likelihood of being Se deficient.

## 4. Discussion

### 4.1. Context of the study

Zimbabwe has a high prevalence of MNDs affecting more than 50% of the population (44). This estimate is from a decade ago and based on anaemia prevalence only. However, anaemia is not synonymous with MNDs (1) since it can be caused by factors unrelated to micronutrient status (61). Direct indicators of MNDs (e.g., biomarkers of Fe, I, Se, and Zn status); are excluded in ZDHS due to cost and gaps in research capacity, such that the last national micronutrient survey was conducted in 2012 (62). Thus, the country does not have current micronutrient data. Additionally, Zimbabwe population studies have been limited to a few micronutrients namely, vitamin A, I, Zn, Fe, and folate (62) as such, data on Se is not nationally available.

### 4.2. Overview of the current study and summary of the findings

The study sought to determine the prevalence, geospatial patterns, and distribution of Se deficiency in WRA and children aged 6–59 months in Mutasa, Shamva, and Murewa. The proportionate representation of participants from the districts allowed fair comparisons. The prevalence of Se deficiency was high and widespread in all three districts. Thus, we contemplate that Se deficiency is potentially a public health issue of national relevance. Selenium deficiency was more prevalent in children compared to women. Variations in Se concentrations were notable across districts, with Murewa having the highest prevalence of Se deficiency. The variations in plasma Se concentration between districts than within districts show that Se status is under strong geospatial control (24). Controls on Se status were driven by short-range spatial variation for WRA and children aged 6–59 months residing in Murewa, Shamva, and Mutasa districts.

### 4.3. Prevalence of Se deficiency in WRA and children 6–59 months in rural Zimbabwe

The median values of plasma Se concentration in women are similar to those established in other African countries (33), including Malawi and Ethiopia. However, the median plasma Se concentrations in children are low compared to China, a known Se-deficient country reporting levels as low as <40 μg/L (63). Globally, average plasma Se concentrations fall within 80–120 μg/L (23), showing the magnitude of Se deficiency risk among the Zimbabwean population. Different thresholds of adequacy reflect the expression of essential selenoproteins and the hierarchy of biological functions (15), whereby expression of SELENOP, GPX3, and IDI are maximal at plasma Se concentrations of >100, >84.9, and >64.8 μg/L (10), respectively. Recent evidence from laboratory and epidemiologic studies has shed a different light on Se’s health effects and its recommended range of environmental exposure compared with earlier research. Adverse effects (type 2 diabetes) have been reported in Western populations at low Se exposure levels, sometimes below or slightly above Se intakes needed to maximize selenoprotein expression and activity (17). The new evidence indicates the need to reassess Se dietary reference values and upper intake level (17).

Based on earlier research Se adequacy cutoff points, our findings suggest that Se deficiency may lead to an increased risk of oxidative stress among most women and children in our study area, and an increased risk of thyroid dysfunction among most children and approximately half of the women. Widespread Se deficiencies, particularly in rural areas, have been reported in Malawi (24) and Ethiopia (23, 38).

The findings of high Se deficiency prevalence are not surprising considering similarities in geography, diet, and socioeconomic conditions between Zimbabwe and Malawi. In both countries, rural populations rely on highly localised agriculture and food systems, where dietary intakes are supplied through subsistence production and locally based food purchases (64–66). When these localised food systems occur in low Se environments, the entry of Se into diets is limited. To compound matters, Zimbabwe has limited access to freshwater fish, which are an essential source of Se (and many other micronutrients) as observed in Malawi (24). Our current findings indicate Se deficiency is likely widespread in Zimbabwe, with a high risk of severe Se deficiency. Therefore, there is a pressing need to conduct surveillance work in other regions and demographic groups. A nationally representative biomarker survey is recommended to establish the magnitude of the problem based on revised cutoff points as suggested by the new evidence (17).

### 4.4. Geospatial modeling of Se deficiency in WRA and children resident in Murewa, Shamva, and Mutasa districts

The minimum number of sampling points used for modelling in the current study was sufficient to avoid bias. Effective variogram estimations require a minimum of 100–150 points (54). In the current study, most of the experimental variograms show variation over short distances up to only 12 km, so the maps appear very “patchy” with similar values in the clusters of observations. This suggests that, in these locations and demographic groups, controls on Se status are driven by short-range variation and are therefore not showing marked spatial trends within districts (54, 59). Selenium status was reasonably homogenous within each district. However, there was substantial inter-district variation, suggesting that there are marked spatial patterns when the sampling area is scaled up; as reflected in other African countries; Côte d’Ivoire (67, 68), Ethiopia (23) and Malawi (24). In Ethiopia spatial factors influencing Se status operated at distances up to 200 km (23) whilst in Malawi spatial dependence was observed at up to 120 km (24). Variations might be explained by the scale of the studies, being a pilot, the current study was just a snapshot limited to district level. Thus, it had a smaller data frame compared to the Ethiopian and Malawian studies that were conducted at a national scale, with sampling points dotted around a larger area which improves the accuracy of the empirical variograms (54). The variation in plasma Se concentration between the districts could also suggest the influence of other spatial parameters, such as soil properties, climate, and landscape (29, 36). Therefore, resource endowment for further investigations may be important to continue to build an evidence base, through complementary surveillance of soils, crops, and biomarkers. Predictions have an attached uncertainty (60) and hence, no map is perfect (69). Nevertheless, the results of the current study predicted a high risk of Se deficiency in both children and WRA residing in the three districts. The maps and the underlying statistical model generated in the current study could be useful to plan further investigations that identify other spatial parameters contributing to the marked between-district variation in the Se status of women and children.

### 4.5. Limitations of study

The current study is limited in geographic scope and we are not able to infer Se status or risk of deficiency across Zimbabwe, particularly since Se status is likely to be under strong geographic control, as observed in other contexts. There is no consensus on the most appropriate threshold to indicate Se deficiency risk. The current study only measured plasma Se concentration which was used as a proxy indicator for the status of the secondary biomarkers (SELENOP, GPX3, and IDI), and thus may not be truly reflective of the selenoprotein status. Additional direct measurements of selenoproteins would complement the findings of the current study. Nevertheless, the present study contributes to the body of knowledge on the application of geostatistical modeling for the interpolation and mapping of nutrient deficiencies.

## 5. Conclusion

The current study showed that the prevalence of Se deficiency was widespread among children aged 6–59 months and WRA in the three pilot districts. Spatial controls on Se status in WRA and children from these districts were driven by short-range variation. There was no marked spatial pattern within districts but a substantial inter-district variation. The prevalence of Se deficiency and the very low concentrations of plasma Se concentrations recorded in the select districts is cause for concern. Further investigations at the population level are warranted to measure the magnitude of the problem using reassessed Se dietary reference values and upper intake level as suggested by new evidence and to build more evidence base to substantiate the current findings and followed by the mapping of strategies that can be used to reduce Se deficiency at the population level.

## Data availability statement

The raw data supporting the conclusions of this article will be made available by the authors, without undue reservation.

## Ethics statement

The studies involving human participants were reviewed and approved by Institutional Review Boards (IRBs) of the University of Nottingham (Reference#446-1912) and the Medical Research Council of Zimbabwe (MRCZ/A/2575 & MRCZ/A/2664). Written informed consent to participate in this study was provided by the participants’ legal guardian/next of kin.

## Author contributions

BM was the lead in the conceptualization, data curation, formal analysis, and writing of the original draft. PC and TM contributed to study conceptualization, lead supervision, reviewing, and editing of the draft paper. CC, MD, EJ, MM-K, HN, TN, and AK supported the conceptualization, data curation, and validation, and reviewed and edited the draft paper. RL, EB, and MB contributed to the study conceptualization, data curation, formal analysis, writing, and review of the paper. All authors contributed to the article and approved the submitted version.

## Funding

The authors acknowledge funding from the UK Research and Innovation (UKRI) Global Challenges Research Fund (GCRF) (grant number EP/T015667/1); “Translating GeoNutrition: Reducing mineral micronutrient deficiencies (MMNDs) in Zimbabwe.” The work was also supported in part by Bill & Melinda Gates Foundation grant INV-009129 through the GeoNutrition project. Under the grant conditions of the Foundation, a Creative Commons Attribution 4.0 Generic License has already been assigned to the Author’s Accepted Manuscript version that might arise from this submission. The funder had no role in the design, execution, analysis, or interpretation of the data.

## Acknowledgments

The authors thank all the parents and caregivers of the infants for participating in the study. Gratitude to the Ministry of Health and Child Care (MoHCC) authorities for their collaboration and support; ZIMSTAT for the household listing and mapping exercise and advice provided by GeoNutrition researchers.

## Conflict of interest

The authors declare that the research was conducted in the absence of any commercial or financial relationships that could be construed as a potential conflict of interest.

## Publisher’s note

All claims expressed in this article are solely those of the authors and do not necessarily represent those of their affiliated organizations, or those of the publisher, the editors and the reviewers. Any product that may be evaluated in this article, or claim that may be made by its manufacturer, is not guaranteed or endorsed by the publisher.

## References

[1] Stevens GA Beal T Mbuya MNN Luo H Neufeld LM Addo OY. Micronutrient deficiencies among preschool-aged children and women of reproductive age worldwide: a pooled analysis of individual-level data from population-representative surveys. Lancet Glob Health. (2022) 10:e1590–9. doi: 10.1016/S2214-109X(22)00367-9, PMID: 36240826 PMC10918648

[2] Kumssa DB Joy EJM Ander EL Watts MJ Young SD Walker S. Dietary calcium, and zinc deficiency risks are decreasing but remain prevalent. Sci Rep. (2015) 5:10974. doi: 10.1038/srep10974 26098577 PMC4476434

[3] Willett W Rockström J Loken B Springmann M Lang T Vermeulen S. Food in the Anthropocene: The EAT-lancet commission on healthy diets from sustainable food systems. Lancet. (2019) 393:447–92. doi: 10.1016/S0140-6736(18)31788-4 30660336

[4] Joy EJM Kumssa DB Broadley MR Watts MJ Young SD Chilimba ADC. Dietary mineral supplies in Malawi: spatial and socioeconomic assessment. BMC Nutr. (2015) 1:76. doi: 10.1186/s40795-015-0036-4

[5] Wang J Wang H Chang S Zhao L Fu P Yu W. The influence of malnutrition and micronutrient status on anemic risk in children under 3 years old in poor areas in China. PLoS One. (2015) 10:e0140840. doi: 10.1371/journal.pone.0140840 26488490 PMC4619061

[6] Reich HJ Hondal RJ. Why nature chose selenium. ACS Chem Biol. (2016) 11:821–41. doi: 10.1021/acschembio.6b00031, PMID: 26949981

[7] Rayman MP. The importance of selenium to human health. Lancet. (2000) 356:233–41. doi: 10.1016/S0140-6736(00)02490-9 10963212

[8] Rayman MP. Selenium and human health. Lancet. (2012) 379:1256–68. doi: 10.1016/S0140-6736(11)61452-9 22381456

[9] European Food Safety Authority (EFSA). Scientific opinion on dietary reference values for selenium. EFSA J. (2014) 12:3846. doi: 10.2903/j.efsa.2014.3846 PMC13136995 42087966

[10] Thomson CD. Assessment of requirements for selenium and adequacy of selenium status: a review. Eur J Clin Nutr. (2004) 58:391–402. doi: 10.1038/sj.ejcn.1601800, PMID: 14985676

[11] Fairweather-Tait SJ Collings R Hurst R. Selenium bioavailability: current knowledge and future research requirements. Am J Clin Nutr. (2010) 91:1484S–91S. doi: 10.3945/ajcn.2010.28674J 20200264

[12] Surai PF Kochish II Fisinin VI Juniper DT. Revisiting oxidative stress and the use of organic selenium in dairy cow nutrition. Animals. (2019) 9:462. doi: 10.3390/ani9070462, PMID: 31331084 PMC6680431

[13] Fairweather-Tait SJ Bao Y Broadley MR Collings R Ford D Hesketh JE. Selenium in human health and disease. Antioxid Redox Signal. (2011) 14:1337–83. doi: 10.1089/ars.2010.3275 20812787

[14] World Health Organization (WHO). Selenium in drinking-water. In background document for preparation of WHO guidelines for drinking-water quality. Geneva, Switzerland: World Health Organization (WHO/SDE/WSH/0304/13) (2003).

[15] Combs GF. Biomarkers of selenium status. Nutrients. (2015) 7:2209–36. doi: 10.3390/nu7042209, PMID: 25835046 PMC4425141

[16] Pieczyńska J Grajeta H. The role of selenium in human conception and pregnancy. J Trace Elem Med Biol. (2015) 29:31–8. doi: 10.1016/j.jtemb.2014.07.003 25175508

[17] Vinceti M Filippini T Jablonska E Saito Y Wise LA. Safety of selenium exposure and limitations of selenoprotein maximization: molecular and epidemiologic perspectives. Environ Res. (2022) 211:113092. doi: 10.1016/j.envres.2022, PMID: 35259406

[18] World Health Organization (WHO). WHO|trace elements in human nutrition and health. Geneva, Switzerland: WHO (2016).

[19] Barchielli G Capperucci A Tanini D. The role of selenium in pathologies: an updated review. Antioxidants. (2022) 11:251. doi: 10.3390/antiox11020251, PMID: 35204134 PMC8868242

[20] Institute of Medicine (IOM). Dietary reference intakes: applications in dietary assessment. Washington, DC: Natl Acad Press (2000).

[21] Institute of Medicine (IOM). Dietary reference intakes (DRIs): recommended dietary allowances and adequate intakes. Washington, DC: IOM (2010).

[22] Rayman MP Infante HG Sargent M. Food-chain selenium, and human health: spotlight on speciation. Br J Nutr. (2008) 100:238–53. doi: 10.1017/S0007114508922522, PMID: 18346307

[23] Belay A Joy EJM Chagumaira C Zerfu D Ander EL Young SD. Selenium deficiency is widespread and spatially dependent in Ethiopia. Nutrients. (2020) 12:1–17. doi: 10.3390/nu12061565, PMID: 32471236 PMC7353016

[24] Phiri FP Ander EL Bailey EH Chilima B Chilimba ADC Gondwe J. The risk of selenium deficiency in Malawi is large and varies over multiple spatial scales. Sci Rep. (2019) 9:6566. doi: 10.1038/s41598-019-43013-z, PMID: 31024041 PMC6484074

[25] Burk RF Norsworthy BK Hill KEMA Motley AK Byrne DW. Effects of chemical form of selenium on plasma biomarkers in a high-dose human supplementation trial. Cancer Epidemiol Biomark Prev. (2006) 15:804–10. doi: 10.1158/1055-9965.EPI-05-0950 16614127

[26] Tóth RJ Csapó J. The role of selenium in nutrition – a review. Acta Univ Sapientiae, Aliment. (2018) 11:128–44. doi: 10.2478/ausal-2018-0008

[27] Guillin OM Vindry C Ohlmann T Chavatte L. Selenium, selenoproteins and viral infection. Nutrients. (2019) 11:1–33. doi: 10.3390/nu11092101, PMID: 31487871 PMC6769590

[28] World Health Organization (WHO). HIV/AIDS. Geneva, Switzerland: WHO (2018).

[29] Fordyce FM. Selenium deficiency and toxicity in the environment. Essentials of medical geology: Revised. (2013). 375–416. doi: 10.1007/978-94-007-4375-5_16

[30] Kieliszek M Błażejak S. Selenium: significance, and outlook for supplementation. Nutrition. (2013) 29:713–8. doi: 10.1016/j.nut.2012.11.012, PMID: 23422539

[31] Gashu D Nalivata PC Amede T Ander EL Bailey EH Botoman L. The nutritional quality of cereals varies geospatially in Ethiopia and Malawi. Nature. (2021) 594:71–6. doi: 10.1038/s41586-021-03559-3, PMID: 34012114 PMC8172382

[32] Jones GD Droz B Greve P Gottschalk P Poffet D. Selenium deficiency risk predicted to increase under future climate change. Proc Natl Acad Sci U S A. (2017) 114:2848–53. doi: 10.1073/pnas.16115761142017;114(11), PMID: 28223487 PMC5358348

[33] Ligowe IS Phiri FP Ander EL Bailey EH Chilimba ADC Gashu D. Selenium deficiency risks in sub-Saharan African food systems and their geospatial linkages. NS. (2020) 79:457–67. doi: 10.1017/S0029665120006904, PMID: 32264979

[34] Mutonhodza B Joy EJM Bailey EH Lark MR Kangara MGM Broadley MR. Linkages between soil, crop, livestock, and human selenium status in sub-Saharan Africa: a scoping review. Int J Food Sci Technol. (2022) 57:6336–49. doi: 10.1111/ijfs.15979, PMID: 36605250 PMC9804181

[35] Joy EJM Ander EL Young SD Black CR Watts MJ Chilimba ADC. Dietary mineral supplies in Africa. Physiol Plant. (2014) 151:208–29. doi: 10.1111/ppl.12144, PMID: 24524331 PMC4235459

[36] Tolu J Bouchet S Helfenstein J Hausheer O Chékifi S Frossard E. Understanding soil selenium accumulation and bioavailability through size resolved and elemental characterization of soil extracts. Nat Commun. (2022) 13:6974. doi: 10.1038/s41467-022-34731-6, PMID: 36379945 PMC9666626

[37] Stefanowicz FA Talwar D O’Reilly DSJ Dickinson N Atkinson J Hursthouse AS. Erythrocyte selenium concentration as a marker of selenium status. Clin Nutr. (2013) 32:837–42. doi: 10.1016/j.clnu.2013.01.005, PMID: 23391458

[38] Gashu D Marquis GS Bougma K Stoecker BJ. Spatial variation of human selenium in Ethiopia. Biol Trace Elem Res. (2018) 189:354–60. doi: 10.1007/s12011-018-1489-5, PMID: 30167960

[39] Fordyce FM Masara D Appleton JD. British Geological Survey technical report WC/94/3 overseas geology series final report on stream sediment, soil and forage chemistry as indicators of cattle mineral status in north-East Zimbabwe. A Report prepared for the Overseas Development Administrat. United Kingdom: British Geological Survey (BGS) (1994).

[40] Mpofu IDT Ndlovu LR Casey NH. The copper, cobalt, iron, selenium, and zinc status of cattle in the Sanyati and Chinamhora smallholder grazing areas of Zimbabwe. Asian-Australasian J Anim Sci. (1999) 12:579–84. doi: 10.5713/ajas.1999.579

[41] Kuona P Mashavave G Kandawasvika GQ Dzangare J Masanganise M Chandiwana P. Plasma selenium levels and nutritional status of school children from an HIV prevention programme in Zimbabwe. J Trop Dis. (2014) 02:1–7. doi: 10.4172/2329-891x

[42] Zimbabwe Ministry of Health and Child Welfare. Zimbabwe National Nutrition Survey – 2010. Harare, Zimbabwe: Nutrition (2010).

[43] Ministry of Health and Child Care (MoHCC). Zimbabwe National Nutrition Survey 2018. Harare, Zimbabwe: Nutrition. 2018 p.

[44] Zimbabwe National Statistics Agency (ZIMSTAT). Zimbabwe demographic and health survey. Zimbabwe demographic and health survey 2015. Harare, Zimbabwe. (2015)

[45] Zimbabwe National Statistics Agency (ZIMSTAT). Zimbabwe population census. Population census office. Zimbabwe: Harare (2012). 2012 p.

[46] Jabkowski P. A meta-analysis of within-household selection impact on survey outcome rates, demographic representation, and sample quality in the European social survey. Ask Res Methods. (2017) 26:31–60. doi: 10.18061/1811/81932

[47] Centres for Disease Control and Prevention (CDC). Mapping public health scenario. Contouring Human Dev. (2020) 59:1–10. doi: 10.1007/978-981-15-4083-7_7

[48] World Health Organization (WHO). Micronutrient survey manual. Geneva, Switzerland: WHO (2020).

[49] Centres for Disease Control and Prevention (CDC). A quick-reference tool for hemolysis status. May. 2021. Prevention C for DC and. Mapping public health [internet]. CDC MUSEUM 2020. p. 1–10. Available at: https://www.cdc.gov/museum/pdf/cdcm-pha-stem-mapping-public-health-lesson.pdf.

[50] Suchdev PS Namaste SML Aaron GJ Raiten DJ Brown KH Flores-Ayala R. Overview of the biomarkers reflecting inflammation and nutritional determinants of anemia (BRINDA) project. Adv Nutr. (2016) 7:349–56. doi: 10.3945/an.115.010215, PMID: 26980818 PMC4785469

[51] Team R core. R: The R project for statistical computing. R foundation Vienna, Austria: Foundation for Statistical Computing (2023).

[52] Chagumaira C Chimungu JG Gashu D Nalivata PC Broadley MR Milne AE. Communicating uncertainties in spatial predictions of grain micronutrient concentration. Geosci Commun. (2021) 4:245–65. doi: 10.5194/gc-4-245-2021

[53] Diggle P Ribeiro PJ. Model-based geostatistics. New York: Springer-Verlag (2010).

[54] Oliver MA Webster R. A tutorial guide to geostatistics: computing and modelling variograms and kriging. Catena. (2014) 113:56–69. doi: 10.1016/j.catena.2013.09.006

[55] Lark RM. A comparison of some robust estimators of the variogram for use in soil survey. Eur J Soil Sci. (2000) 51:137–57. doi: 10.1046/j.1365-2389.2000.00280.x

[56] Rosen BP Liu Z. Transport pathways for arsenic and selenium: a minireview. Environ Int. (2009) 35:512–5. doi: 10.1016/j.envint.2008.07.023, PMID: 18789529 PMC2719050

[57] Mastrandrea MD Mach KJ Plattner GK Edenhofer O Stocker TF Field CB. The IPCC AR5 guidance note on consistent treatment of uncertainties: a common approach across the working groups. Clim Chang. (2011) 108:675–91. doi: 10.1007/s10584-011-0178-6

[58] Cressie N Hawkins DM. Robust estimation of the variogram. Math Geol. (1980) 12:115–25. doi: 10.1007/BF01035243

[59] Rawlins Barry Brown Sarah. Assessing geostatistical methods for presenting urban soil geochemical data from Coventry. British Geological Survey Internal Report, IR/03/012. United Kingdom: British Geological Survey (BGS) (2003) 31.

[60] Chagumaira C. Geospatial modelling of soil geochemistry at national-scale for improved human nutrition thesis submitted to the University of Nottingham for the degree of doctor of philosophy. 2022. Tóth RJ Csapó J. (Eds.) The role of selenium in nutrition – A review. Acta Univ Sapientiae, Aliment. (2018);11:128–144

[61] Chaparro CM Suchdev PS. Anemia epidemiology, pathophysiology, and etiology in low- and middle-income countries. Ann N Y Acad Sci. (2019) 1450:15–31. doi: 10.1111/NYAS.14092, PMID: 31008520 PMC6697587

[62] Ministry of Health and Child Welfare. Zimbabwe National Micronutrient Survey Results, 2012. Zimbabwe: Harare (2015).

[63] Li N Gao Z Luo D Tang X Chen D Hu Y. Selenium level in the environment and the population of Zhoukoudian area, Beijing, China. Sci Total Environ. (2007) 381:105–11. doi: 10.1016/j.scitotenv.2007.03.027, PMID: 17509665

[64] Malawi National Statistics Office (MNSO). Malawi third integrated household survey (IHS3). MNSO (2012), 2010–2011.

[65] Zimbabwe National Statistics Agency (ZIMSTAT). Consumption and expenditure survey 2017 report. Harare, Zimbabwe: ZIMSTAT (2018).

[66] Zimbabwe Vulnerability Assessment Committee (ZIMVAC). Zimbabwe rural livelihood baseline profiles. Harare, Zimbabwe: ZIMVAC (2011) 1–97.

[67] Arnaud J Malvy D Richard MJ Faure H Chaventré A. Selenium status in an iodine deficient population of the West Ivory Coast. J Physiol Anthropol Appl Hum Sci. (2001) 20:81–4. doi: 10.2114/jpa.20.81 11385942

[68] Tiahou G Maire B Dupuy A Delage M Vernet MH Mathieu-Daudé JC. Lack of oxidative stress in a selenium deficient area in Ivory Coast: potential nutritional antioxidant role of crude palm oil. Eur J Nutr. (2004) 43:367–74. doi: 10.1007/s00394-004-0484-0 15490200

[69] Heuvelink GBM. Uncertainty and uncertainty propagation in soil mapping and modelling. Cham: Springer (2018) 439–461. doi: 10.1007/978-3-319-63439-5_141

[70] Mutonhodza B Dembedza MP Murray LR Joy EJM Manzeke-Kangara MG Njovo H. Anemia in children aged 6–59 months was significantly associated with maternal anemia status in rural Zimbabwe. Food Sci Nutr. (2022) 11:1232–46. doi: 10.1002/fsn3.3157, PMID: 36911837 PMC10003031

[71] Erhardt JG Estes JE Pfeiffer CM Biesalski HK Craft NE. Combined measurement of ferritin, soluble transferrin receptor, retinol binding protein, and C-reactive protein by an inexpensive, sensitive, and simple sandwich enzyme-linked immunosorbent assay technique. J Nutr. (2004) 134:3127–32. doi: 10.1093/jn/134.11.3127 15514286

