# Supplementary material for: A pilot survey of selenium status and its geospatial variation among children and women in three rural districts of Zimbabwe
Source: Front Nutr. 2023 Jul 11;10:1235113. doi: 10.3389/fnut.2023.1235113 (PMC10367098; doi:10.3389/fnut.2023.1235113)
Supplement: Supplementary file 1 [file Data_Sheet_1.pdf]

frontiers

Supplementary Material

A pilot survey of selenium status and its geospatial variation among children and women in three rural districts of Zimbabwe

Beaula Mutonhodza[1*], Christopher Chagumaira[2], Mavis P. Dembedza[1], Edward J.M. Joy[3,4], Muneta G. Manzeke-Kangara[4], Handrea Njovo[5], Tasiana K. Nyadzayo[5], R. Murray Lark[2] , Alexander A. Kalimbira[6], Elizabeth H. Bailey[2*], Martin R. Broadley[4], Tonderayi M. Matsungo[1, †] and Prosper Chopera[1, †]

[1]Department of Nutrition, Dietetics and Food Sciences, University of Zimbabwe. P.O. Box MP167, Mt Pleasant, Harare, Zimbabwe.

[2]School of Biosciences, Sutton Bonington Campus, University of Nottingham, Loughborough, Leicestershire LE12 5RD, United Kingdom.

[3]London School for Hygiene & Tropical Medicine, Keppel Street London, WC1E 7HT, United Kingdom.

[4]Rothamsted Research, West Common, Harpenden, AL5 2JQ, United Kingdom

[5] National Nutrition Unit, Ministry of Health and Child Care of Zimbabwe. Box CY 1122 Causeway, Harare, Zimbabwe.

[6]Department of Human Nutrition and Health, Lilongwe University of Agriculture and Natural Resources, P.O. Box 219, Lilongwe, Malawi.

Short Title: **Distribution of selenium deficiency in Zimbabwe**

***Corresponding Authors:**

	**Names**	**Email address**	**Contact Number**
1	Ms. Beaula Mutonhodza	bmutonhodza@science.uz.ac.zw	+263 (0)772210397
2	Dr Elizabeth Bailey	liz.bailey@nottingham.ac.uk	+44 (0)1159516255

Supplementary material 1:Correlation between plasma Se and acute phase proteins

Variable	Test statistic	Se μg / L
Children 6 - 59 months		
CRP mg / L	**Pearson Correlation**	**0.009**
	Sig. (2-tailed)	**0.817**
	**n**	**693**
AGP g / L	**Pearson Correlation**	**-0.049**
	Sig. (2-tailed)	**0.199**
	**n**	**692**
Women of reproductive age (15 - 49 years)		
CRP mg /L	**Pearson Correlation**	**-0.003**
	Sig. (2-tailed)	**0.937**
	**n**	**829**
AGP g / L	**Pearson Correlation**	**0.029**
	Sig. (2-tailed)	**0.402**
	**n**	**829**

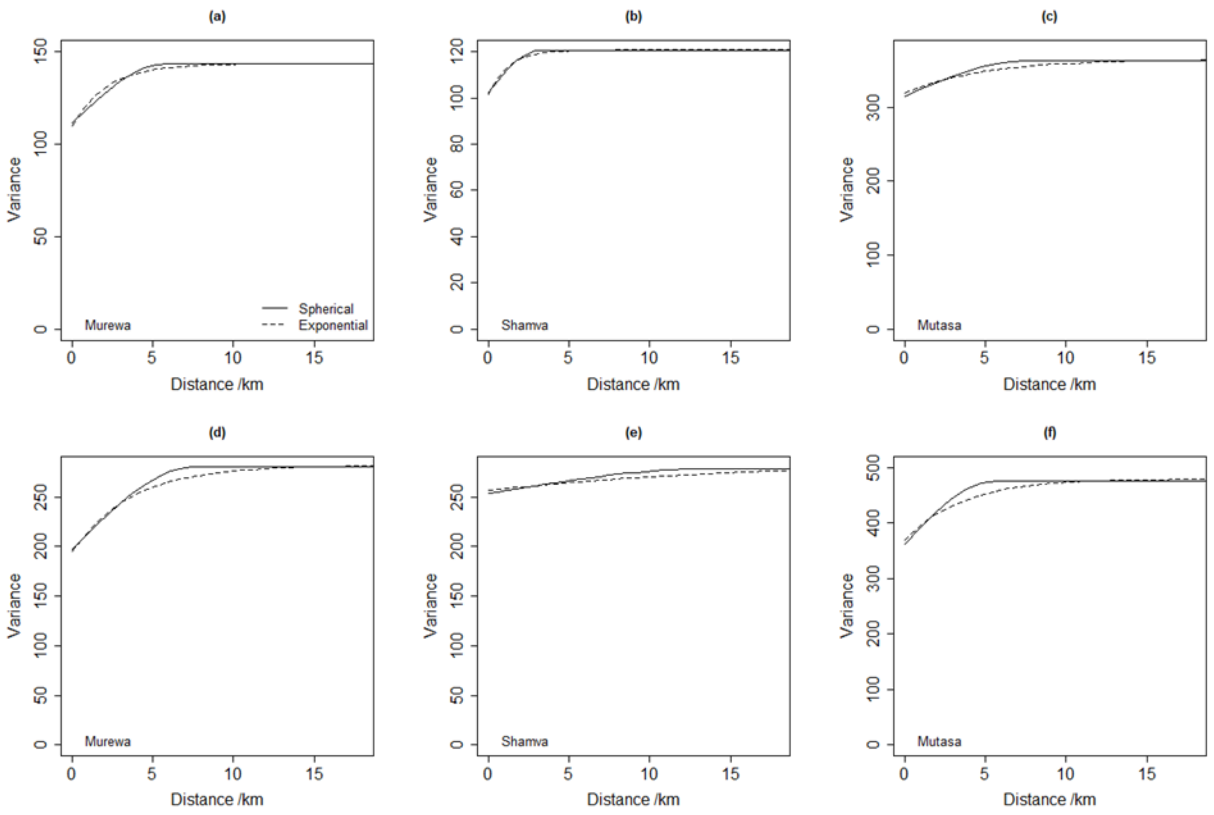

Supplementary material 2: Fitted variogram models for demographic groups; (a), (b), (c) for children 6-59 months and (d), (e), (f) for WRA by district. The models are fitted by residual maximum likelihood (REML) and in each case the exponential model is shown by a dotted line and the spherical model by a solid line.
